# Chemists and Companies

**Published:** 1906-05-26

**Authors:** 


### The Hospital

A Journal of

The Medical Sciences and Hospital Administration.

Vol. XL.—No. 1,026. SATURDAY, MAY 26, 1906.

### Chemists and Companies.

The House of Lords, during the earlier part of the present month, has, in addition to its other activities, been concerned in the discussion of a question which primarily affects pharmacists on the one hand and certain limited companies engaged in the retail sale of drugs on the other. The subject of contention, in brief, has been, whether the use of such terms as "pharmaceutical chemist" and "chemist and druggist" ought to be confined to individuals who have passed the qualifying examinations of the Pharmaceutical Society, or whether they may also be employed for trade purposes by any limited liability company which selects as a manager for the purpose of a drug business a person qualified under the Pharmacy Act. By a small majority their Lordships affirmed the latter of these propositions, and although the Poisons and Pharmacy Bill in which the clause in question occurs has yet to be debated in the House of Commons and may therefore undergo substantial alteration, it is not inappropriate to consider the decision at which one branch of the Legislature has so far arrived. As must be well known to all our readers, the establishment of limited companies for the retail sale of drugs has become a familiar event in recent years, and such companies publicly announce themselves as "chemists" or "dispensing chemists," usually adding to these titles some more or less alluring qualification for commercial or advertising ends. This practice has been attacked in the law courts, but without success, and it must be accepted that it is at present competent for any limited liability company not only to conduct the business of a chemist and druggist, but also to use the corresponding titles, provided only that a qualified manager is placed in charge of each of the shops which the company owns. None of the directors need be a qualified chemist, and the company may be one of the "family" or "one-man" order, so that what is illegal for each of seven individuals acting separately becomes legal when the same seven persons register themselves as a limited liability company. That, on the face of it is a somewhat extraordinary position, and it is hardly a matter for wonder that chemists and druggists, whose financial interests are involved, should endeavour to alter it. They urge that the titles which they bear are bestowed with the authority of the State and as a guarantee of personal fitness to accept and discharge the not inconsiderable responsibilities of the calling of a dispenser and pharmacist, and further, that the acquisition of these titles has involved each one of them in an expenditure of time, energy, and money. They now see the law, which excludes unqualified individuals from using these designations, permitting collections of individuals in the form of limited companies to employ them. Certainly there does seem a substantial injustice in this procedure, and on this ground, apart for the moment from others, we think the chemists are entitled to sympathy. But the principle involved is even of more importance than this individual application of it. If it may be applied to pharmacy it seems difficult to prevent its extension to other callings and professions, such, for example, as medicine or dentistry or teaching; for if the directors of a company may in their collective capacity describe themselves as chemists, though no one of their number possesses any such qualification, why may they not also describe themselves as physicians, or surgeons, or dentists, or masters of arts? To the plain man any such proceeding is wanting in straightforwardness and sounds uncommonly like sharp practice. There is, however, another reason why the medical profession should concern itself with the recent decision of the House of Lords, and it is that such a decision is antagonistic to the development of pharmacy as a responsible individual calling. We have no sympathy with any attempt to establish a trade monopoly, but we are strongly of opinion that it is to the interest alike of the medical profession and of the public to encourage the development and culture of those who not only have the necessary technical qualifications, but who, in addition, recognise that in pharmaceutical practice there is a claim for an ethical standard somewhat different from that which regulates the commercial conscience. It is well to have this supported by a sense of individual dignity and responsibility, and we are doubtful whether these conditions will be so readily fulfilled under the concealment of a limited company as in establishments which depend for reputation on personal character as well as on trade equipment. With companies as trading concerns we have no quarrel, but we do hold that chemists should be protected in the use of titles which they have acquired under conditions prescribed by the Legislature, and that the special and personal responsibilities of the pharmacist should be recognised both as a scientific and a public interest.

